# Clinical outcomes of lutetium-177–PSMA-617 in a racially diverse cohort of patients with metastatic castration-resistant prostate cancer

**DOI:** 10.1093/oncolo/oyag022

**Published:** 2026-02-03

**Authors:** Margo Brooke Gerke, Akshay Bedmutha, Angelo Marra, Yuan Liu, Jacqueline T Brown, Bassel Nazha, Jacob E Berchuck, Ravi Bharat Parikh, Shahid Ahmed, Jordan Alana Ciuro, Caitlin Hartman, Greta Russler McClintock, Sarah Caulfield, Omer Kucuk, Bradley Curtis Carthon, David M Schuster, Saima Muzahir, Mehmet Asim Bilen

**Affiliations:** Emory University School of Medicine, Atlanta, GA 30322, United States; Department of Radiology and Imaging Sciences, Division of Nuclear Medicine and Molecular Imaging, Emory University, Atlanta, GA 30322, United States; Biostatistics Shared Resource, Winship Cancer Institute, Emory University, Atlanta, GA 30322, United States; Department of Biostatistics and Bioinformatics, Emory University, Atlanta, GA 30322, United States; Department of Hematology and Medical Oncology, Emory University School of Medicine, Atlanta, GA 30322, United States; Emory Winship Cancer Institute, Atlanta, GA 30322, United States; Piedmont Cancer Institute, Atlanta, GA 30322, United States; Department of Hematology and Medical Oncology, Emory University School of Medicine, Atlanta, GA 30322, United States; Emory Winship Cancer Institute, Atlanta, GA 30322, United States; Department of Hematology and Medical Oncology, Emory University School of Medicine, Atlanta, GA 30322, United States; Emory Winship Cancer Institute, Atlanta, GA 30322, United States; Department of Hematology and Medical Oncology, Emory University School of Medicine, Atlanta, GA 30322, United States; Emory Winship Cancer Institute, Atlanta, GA 30322, United States; Department of Hematology and Medical Oncology, Emory University School of Medicine, Atlanta, GA 30322, United States; Emory Winship Cancer Institute, Atlanta, GA 30322, United States; Department of Hematology and Medical Oncology, Emory University School of Medicine, Atlanta, GA 30322, United States; Emory Winship Cancer Institute, Atlanta, GA 30322, United States; Department of Hematology and Medical Oncology, Emory University School of Medicine, Atlanta, GA 30322, United States; Emory Winship Cancer Institute, Atlanta, GA 30322, United States; Department of Hematology and Medical Oncology, Emory University School of Medicine, Atlanta, GA 30322, United States; Emory Winship Cancer Institute, Atlanta, GA 30322, United States; Department of Hematology and Medical Oncology, Emory University School of Medicine, Atlanta, GA 30322, United States; Emory Winship Cancer Institute, Atlanta, GA 30322, United States; Department of Hematology and Medical Oncology, Emory University School of Medicine, Atlanta, GA 30322, United States; Emory Winship Cancer Institute, Atlanta, GA 30322, United States; Department of Radiology and Imaging Sciences, Division of Nuclear Medicine and Molecular Imaging, Emory University, Atlanta, GA 30322, United States; Department of Radiology and Imaging Sciences, Division of Nuclear Medicine and Molecular Imaging, Emory University, Atlanta, GA 30322, United States; Emory University School of Medicine, Atlanta, GA 30322, United States; Department of Hematology and Medical Oncology, Emory University School of Medicine, Atlanta, GA 30322, United States; Emory Winship Cancer Institute, Atlanta, GA 30322, United States

**Keywords:** radioligand therapy, lutetium-177–PSMA-617, racial disparities, real-world outcomes, metastatic castration-resistant prostate cancer

## Abstract

**Background:**

Lutetium-177 (^177^Lu)–PSMA-617 is a beta-emitting radioligand approved for treatment of metastatic castration-resistant prostate cancer (mCRPC), despite the underrepresentation of Black patients in pivotal trials. We analyzed outcomes of ^177^Lu-PSMA-617 in a racially diverse cohort.

**Methods:**

Retrospective analysis of patients with mCRPC treated with ^177^Lu-PSMA-617 was conducted at the Emory Winship Cancer Institute. Primary outcomes assessed were progression-free survival (PFS), overall survival (OS), and prostate-specific antigen (PSA) reduction ≥50% (PSA50). Cox proportional hazard models were used for univariate and multivariate OS and PFS, and logistic regression was used for PSA50 analysis.

**Results:**

Among 163 patients treated with ^177^Lu–PSMA-617, 97 (59.5%) self-identified as White or other racial groups and 66 (40.5%) self-identified as Black. On univariate analysis, Black patients had comparable OS, PFS, and PSA50 responses to non-Black patients, with a trend toward improved outcomes (OS HR: 0.82, *P* = .446; PFS HR 0.92, *P* = .655; PSA50 OR = 1.79, *P* = .088). Multivariate analysis demonstrated a non-significant prolonged PFS and reduction in mortality risk for Black patients (PFS HR: 0.65, *P* = .106; OS: HR 0.59, HR *P*-value .081). The odds of a PSA50 response were 2.45 times higher for Black patients (OR = 2.45, *P* = .027).

**Conclusions:**

In our racially diverse cohort of patients with mCRPC, Black patients had PFS and OS comparable to non-Black patients, although wide confidence intervals limit definitive conclusions. Black patients had a significantly greater odds of achieving a PSA50 response. Our findings suggest efficacy of ^177^Lu-PSMA-617 among Black patients in real-world settings and underscore the importance of improved representation in prospective studies.

Implications for Practice This study evaluates the efficacy of ^177^Lu–PSMA-617 in the most racially diverse cohort reported to date. Racial representation in pivotal trials that led to the FDA approval of ^177^Lu–PSMA-617 has been limited. Given the documented racial disparities in prostate cancer outcomes, our findings provide real-world evidence supporting the broad efficacy of ^177^Lu–PSMA-617, reinforcing its equitable use in metastatic castration-resistant prostate cancer. This evidence strengthens clinical confidence in ^177^Lu–PSMA-617 use for Black patients.

## Introduction

Despite therapeutic advances, metastatic castration-resistant prostate cancer (mCRPC) remains an incurable disease. While docetaxel and androgen receptor signaling inhibitors (ARSI) improve survival in mCRPC, therapeutic options are limited following disease progression on these agents.^1–4^ Lutetium-177 (^177^Lu)–PSMA-617 is a radioligand that induces DNA damage and subsequent cell death in prostate-specific membrane antigen (PSMA)-expressing cells. The VISION trial demonstrated improved imaging-based progression-free survival (PFS) and overall survival (OS) with ^177^Lu–PSMA-617 treatment for patients with mCRPC who progressed on ARSI and taxane therapies.^5^ In the THERA-P trial, ^177^Lu-PSMA-617 was compared to cabazitaxel in patients with mCRPC who had previously been treated with docetaxel, reporting prolonged PFS but no significant difference in OS.^6^^,^^7^ The PSMAFore trial evaluated ^177^Lu-PSMA-617 effectiveness in taxane-naïve patients, finding prolonged radiographic PFS in patients treated with ^177^Lu-PSMA-617 compared to a change in ARSI.^8^ Current guidelines recommend considering ^177^Lu-PSMA-617 for patients with PSMA-positive disease who have progressed on ARSI, either before or following taxane-based chemotherapy, consistent with FDA indications.^9^

Prostate cancer remains a significant health disparity in the United States, disproportionately impacting Black men, who experience nearly twice the mortality rate of White men.^10^ The influence of race on the treatment efficacy of ^177^Lu-PSMA-617 remains unknown. Evidence suggests, despite Black men with mCRPC experiencing worse survival outcomes, they may have more robust responses to systemic therapy than White men.^11^ The low representation of Black patients in the VISION (6.6%), THERA-P, and PSMAFore trials underscores the need to evaluate the generalizability of trial outcomes to diverse patient populations.^5^ Our study cohort has the highest proportion of patients who self-identify as Black or African American (40.5%) to date. We aim to be the first to report real-world outcomes of ^177^Lu-PSMA-617 in a racially diverse cohort.

## Methods

### Patients and data collection

Medical records were reviewed for patients with mCRPC treated with ^177^Lu-PSMA-617 at Emory Winship Cancer Institute who initiated treatment between July 20, 2022, and June 17, 2025. Institutional Review Board approval was received for retrospective chart review and analysis. We included all patients who had received at least one cycle of ^177^Lu-PSMA-617 treatment. Imaging-based determination of patient eligibility for ^177^Lu-PSMA-617 was based on the VISION criteria as evaluated on standard PSMA Positron Emission Tomography (PET)/Computed Tomography (CT) imaging. Lesion PSMA-positivity was defined as the presence of PSMA uptake more than the liver background in a metastatic lesion of any size in any organ. Lesion PSMA-negativity was defined as PSMA uptake equal to or less than liver in any metastatic lymph node measuring at least 2.5 centimeters in short axis, or in any soft tissue lesion measuring at least 1 centimeter in short axis.^5^ All patients who underwent ^177^Lu-PSMA-617 therapy and were included in this analysis had at least one PSMA-positive lesion and no PSMA-negative lesion on PET/CT imaging.^5^ Data collected from patients’ electronic medical records included: age, self-reported race, Eastern Cooperative Oncology Group (ECOG) performance status, Gleason grade group, high or low volume metastatic disease per CHAARTED Trial criteria, prior lines of therapy, total cycles of ^177^Lu–PSMA-617, progression date, date of death or last follow-up, and PSA decline. CHAARTED criteria for high volume disease were classified as ≥4 bone lesions with at least one outside the axial skeleton or visceral metastasis.^12^ PSA, hemoglobin, testosterone, and alkaline phosphatase (ALP) were collected at baseline and after each treatment cycle of ^177^Lu–PSMA-617. Median annual household income data was obtained by zip code using the US Census Bureau Income by Zip Code Tabulation area map.^13^

Fifty-two pre-treatment F-18 Piflufolastat (Pylarify, Lantheus Holdings, Inc.) PSMA PET/CT scans were included for this analysis. Ga68-labelled PSMA PET/CT scans were not included, as many of these scans were acquired at different outside imaging centers, introducing variations related to unknown injection doses, equipment calibrations, and acquisition protocols. The background liver standardized uptake value (SUV) mean was utilized as the threshold for delineating PSMA-avid tumor burden. LesionID (MIM Software, Inc.) software was used for auto-contouring of the PSMA-avid disease. Two Nuclear Medicine physicians experienced in PSMA PET/CT reading assessed and edited the contours for accuracy. Final whole-body projections, embedded with whole-body SUV mean, SUV maximum, PSMA tumor volume, and total SUV (the product of SUV mean and PSMA volume), were obtained for analysis.

### Statistical analysis

Primary clinical endpoints assessed in this study include PFS, OS, and PSA50. Progression was defined by either a PSA rise of ≥25% and increase of ≥2 ng/mL above the nadir, PSMA PET/CT imaging-based progression, death or hospice care, whichever first occurred and continuously assessed until July 30, 2025.^14^ OS was defined as months from initiation of ^177^Lu–PSMA-617 to death or transition to hospice care, which was continuously evaluated. PSA50 was defined as a reduction in PSA ≥50% from baseline.

Descriptive statistics were performed for self-reported race, Gleason grade, age, number of prior lines of therapy, median number of ^177^Lu–PSMA-617 cycles, median baseline PSA, and median baseline ALP. Chi-Square and analysis of variance (ANOVA) tests were used to assess categorical and continuous variables, respectively. Univariate survival analyses for OS and PFS were performed using Cox proportional hazards models for the entire cohort and by self-reported race. Kaplan-Meier survival curves were used to estimate survival distributions for PFS and OS. Multivariate Cox proportional hazards models were used to evaluate variables associated with PFS and OS. Backwards stepwise selection was applied with a removal threshold of α = .25 to determine variables retained in model. Logistic regression was used for univariate and multivariate PSA50 analysis. Hazard ratios (HR) and 95% confidence intervals (CI) were estimated for each covariate to determine their independent associations with survival outcomes while controlling for the other included covariates. Proportional hazards assumptions were assessed to ensure model validity. The statistical analysis was performed using SAS 9.4.

## Results

### Patient and disease characteristics

Patient demographics and baseline prostate cancer characteristics for our cohort of 163 patients with PSMA PET-positive mCRPC are presented in Table 1. Sixty-six patients (40.5%) self-identified as Black, and 80 (49%) self-identified as White. The median age of our cohort was 73 years (IQR: 65-80 years). Most patients (86%) had high metastatic tumor burden defined by the CHAARTED trial as 4 or more bone metastases, with at least one metastasis outside the axial skeleton, visceral metastasis, or both.^12^ The median baseline PSA for the overall cohort was 68.4 (IQR: 19.18-342) and the median baseline ALP was 102.5 (IQR: 72-175). Black patients had a median baseline PSA of 123 (IQR: 30.5-373.09) and ALP of 103 (IQR: 70-193), compared to a baseline PSA of 48.6 (13.86-229.02) and ALP of 102 (72-170) for White patients and patients of other racial groups (PSA *P* = .063; ALP *P* = .09). The median number of cycles of ^177^Lu-PSMA-617 received was 4 (IQR: 2-6); 52 patients received 6 cycles, 73 patients had disease progression before to 6 cycles, and 8 patients discontinued before 6 cycles due to an individualized treatment decision due to excellent radiographic and PSA responses, 26 patients were scheduled for ^177^Lu-PSMA-617 after the endpoint of our data collection, and 4 patients did not receive 6 cycles due to absence of further follow-up.

**Table 1.** Patient demographics.

Variable	Total *N* = 163
**Patient race (%)**	
** Black**	66 (40.49)
** Asian**	6 (3.68)
** Caucasian**	80 (49.08)
** Hispanic**	3 (1.84)
** Unknown**	8 (4.91)
**Median household income by zip code (%)**	
** $150,000+**	4 (2.45)
** $100,000–$149,999**	44 (26.99)
** $75,000–$99,999**	50 (30.67)
** $50,000–$74,999**	46 (28.22)
** $25,000–$49,999**	18 (11.04)
** Under $25,000**	1 (0.61)
**CHAARTED^a^ trial result (%)**	
** High**	139 (85.8)
** Low**	23 (14.2)
** Missing**	1
**Total Gleason score**	
** Total, *N***	123
** Mean (SD)**	7.97 (1.14)
** Median (Quartile 1-Quartile 3)**	8 (7-9)
** Minimum-maximum**	4-10
***N*, missing**	40
**Gleason grade group (%)**	
** 6-7**	33 (20.25)
** 8**	23 (14.11)
** 9-10**	53 (32.52)
** ≤6**	11 (6.75)
** Unknown^b^**	43 (26.38)
**Patient age**	
** Total, *N***	163
** Mean (SD)**	72.56 (9.69)
** Median (Quartile 1-Quartile 3)**	73 (65-80)
** Minimum-maximum**	47-98
***N*, missing**	0
**Cycle 1 day 1 prostate-specific antigen**	
** Total, *N***	155
** Mean (SD)**	253.99 (374.68)
** Median (Quartile 1-Quartile 3)**	68.37 (19.18-342)
** Minimum-maximum**	0.1-1300
***N*, missing**	8
**Cycle 1 day 1 alkaline phosphatase**	
** Total, *N***	156
** Mean (SD)**	162.74 (169.63)
** Median (Quartile 1-Quartile 3)**	102.5 (72-175)
** Minimum-maximum**	30-1299
***N*, missing**	7
**Number of prior lines of therapy**	
** Total, *N***	160
** Mean (SD)**	4.72 (1.75)
** Median (Quartile 1-Quartile 3)**	4 (3-6)
** Minimum-maximum**	2-10
***N*, missing**	3
**Number of lutetium-177–PSMA-617 cycles**	
** Total, *N***	163
** Mean (SD)**	3.74 (1.86)
** Median (Quartile 1-Quartile 3)**	4 (2-6)
** Minimum-maximum**	1-6
***N*, missing**	0
**Novel hormonal agents before lutetium-177–PSMA-617 (%)**	
** Used prior to lutetium-177–PSMA-617**	163 (100)
**Prior taxane treatment (%)**	
** No**	25 (16.13)
** Yes**	130 (83.87)
** Missing**	8
**Number of metastases before lutetium-177–PSMA-617**	
** Total, *N***	157
** Mean (SD)**	1.95 (0.97)
** Median (Quartile 1-Quartile 3)**	2 (1-2)
** Minimum-maximum**	1-5
***N*, missing**	6
**Number of bone lesions at initiation (%)**	
** 1-19**	30 (19.61)
** >20**	123 (80.39)
** Missing**	10
**ECOG** ^c^ **at time of starting lutetium-177–PSMA-617 (%)**	
** 0-1**	103 (65.19)
** 2+**	55 (34.81)
** Missing**	5
**Adverse event grade (%)**	
** Adverse event grade < 3**	126 (81.82)
** Adverse event grade 3**	28 (18.18)
** Missing**	9

a CHAARTED criteria defined as 4 or more bone lesions, with at least one outside the skull, vertebral column, ribs, and sternum; or the presence of visceral metastasis.

b For patients with de novo metastatic disease, Gleason score was frequently unavailable due to diagnostic confirmation at a metastatic site.

c Eastern Cooperative Oncology Group performance status.

The SUV maximum, SUV mean, total SUV, and total tumor volume (mL) were obtained from baseline PSMA PET scans, presented in Table 2. The Black cohort had a non-significant higher baseline median SUV maximum of 69.71 (IQR: 42.16-145.94) compared to the median SUV maximum of 43.39 in the non-Black cohort (IQR: 31.47–63.5; *P* = .157). The median SUV mean in the Black cohort was 9.84 (IQR: 8.64-19.57) compared to 10.13 (IQR: 7.27-11.88) in the non-Black cohort (*P* = .215). The total SUV was significantly greater for Black patients compared to patients who identified as White or other racial groups (178,964 vs. 54,012, *P* = .027). The median baseline total tumor volume was greater in the Black cohort, at 346.82 mL (IQR: 118.8-1,372.8), compared to the White or other racial groups cohort, at 120.96 mL (IQR: 49.47-711.87, *P* = .303).

**Table 2.** Standardized uptake values and total tumor volume (mL) in Black versus non-Black patients.

				Racial group		
		
Covariate	Statistics	Level	Total *N* = 44 (100%)	Black *N* = 26 (59.09%)	Non-Black *N* = 18 (40.91%)	*P*-value^a^
**Maximum standardized uptake value (SUVmax)**	Mean (SD)		91.27 (107.9)	110.54 (119.28)	63.45 (84.48)	.157
	Median (Q1-Q3)		49.92 (32.73-85.61)	69.71 (42.16-145.94)	43.39 (31.47-63.5)	
	Minimum—Maximum		18.68-592.01	25.12-592.01	18.68-394.18	
	*N*, missing		0	0	0	
**Mean standardized uptake value**	Mean (SD)		12.74 (7.31)	13.89 (7.48)	11.08 (6.93)	.215
	Median (Q1-Q3)		9.92 (8.59-14.03)	9.84 (8.64-19.57)	10.13 (7.27-11.88)	
	Minimum—Maximum		5.66-37.22	6.12-34.55	5.66-37.22	
	*N*, missing		0	0	0	
**Total standardized uptake value**	Mean (SD)		599475.01 (1101345.25)	900864.59 (1345846.37)	164134.52 (254566.03)	**.027** ^*^
	Median (Q1-Q3)		96707.59 (34479.33-714076.71)	178964.02 (60676-1251897.36)	54011.73 (21451.64-176169.84)	
	Minimum—Maximum		4707.77-4664535.52	15236.23-4664535.52	4707.77-937885.91	
	*N*, missing		0	0	0	
**Total tumor volume (mL)**	Mean (SD)		840.34 (1269.75)	1006.38 (1393.47)	600.5 (1058.07)	.303
	Median (Q1-Q3)		197.92 (97.91-963.22)	346.82 (118.85-1372.8)	120.96 (49.47-711.87)	
	Minimum—Maximum		4.21-4594.47	10.45-4594.47	4.21-3932.81	
	*N*, missing		0	0	0	

a The parametric *P*-value is calculated by ANOVA.

* Black patients had significantly higher total standardized uptake value when compared to non-Black patients.

### Prostate-specific antigen response

In the total study sample, 83 patients (55%) achieved a PSA50 response. PSA50 responses were numerically higher for Black patients (63.5%) compared to non-Black patients (49%; *P* = .087). On univariate analysis, Black patients had a non-significant trend toward greater odds of a PSA50 response (OR = 1.79, 95% CI, 0.92-3.45, *P* = .088), displayed in Table S1. After backward selection with an alpha of 0.25, the following variables were removed from the PSA50 multivariate model: ECOG at time of initiation of ^177^Lu-PSMA-617, Gleason Grade group, number of bone lesions at initiation of ^177^Lu-PSMA-617, and number of prior lines of therapy. The odds of a PSA50 response were 2.45 times higher for Black patients compared to those of non-Black patients (OR: 2.45, 95% CI, 1.11-5.42, *P* = .027) when controlling for median household income by zip code, CHAARTED trial high or low volume disease, prior taxane treatment, number of metastases before treatment, ECOG status, and baseline body mass index, displayed in Table 3. Odds of a PSA50 response were over 3 times greater for patients in the $50,000 to $74,999 income bracket in comparison to the reference group on univariate and multivariate analysis (UVA: OR = 3.80, 95% CI, 1.19-12.14, *P* = .024; MVA OR: 3.2, 95% CI, 1.15-8.87, *P* = .025).

**Table 3.** Multivariate logistic regression of prostate-specific antigen (PSA) response of 50% or more from baseline levels.

			PSA decline ≥50 = Yes		
	
Covariate	Level	*N* ^a^	Odds ratio (95% CI)	OR *P*-value	Type 3 *P*-value
**Racial group**	Black	60	2.45 (1.11-5.42)	.027^*^	**.027** ^*^
	White/other racial groups	77	-	-	
**Median household income by zip code**	$0-$49,000	15	0.52 (0.13-2.09)	.358	**.049** ^***^
	$100,000-$150,000+	40	1.59 (0.63-4.02)	.330	
	$50,000-$74, 999	37	3.20 (1.15-8.87)	**.025** ^**^	
	$75, 000-$99, 999	45	-	-	
**Patient age**		137	1.04 (1.00-1.09)	.060	.060
**CHAARTED trial result**	High	118	0.37 (0.11-1.19)	.095	.095
	Low	19	-	-	
**Taxane treatment**	No	22	2.00 (0.64-6.20)	.231	.231
	Yes	115	-	-	
**Number of metastases before lutetium-177–PSMA-617**		137	0.72 (0.47-1.11)	.141	.141
**Cycle 1 body mass index**		137	1.06 (0.98-1.16)	.169	.169

a Number of observations in the original data set = 150. Number of observations used = 137.

* Black patients had higher odds of a PSA50 response compared to patients of other races.

** Patients with an income of $50,000-74,000 had higher odds of a PSA50 response in comparison to the reference group.

*** Median household income was associated with the odds of a PSA50 response (type 3 *P* = 0.049).

### Progression-free survival

**Figure 1.**
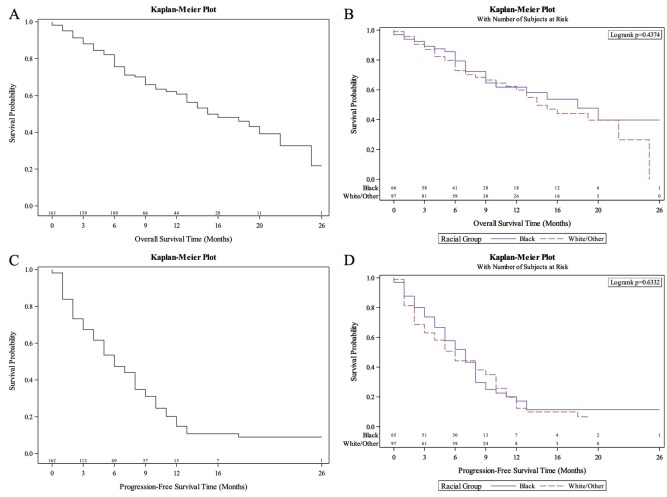
(A) Survival curve for all patients (B) Survival curve for Black patients compared to non-Black patients (C) Progression-free survival for all patients (D) Progression-free survival for Black compared to non-Black patients.

Median PFS for the entire cohort was 6 months (95% CI, 5-8). The 6-month PFS was 47.3% (95% CI, 39%-55.2%) and the 12-month PFS was 14.8% (95% CI, 8.5%-22.7%), displayed in Figure 1C. After backward selection with an alpha level of removal of 0.25, the following variables were removed from multivariable analysis: patient age, body mass index, median household income by zip-code, and prior taxane treatment. On univariate and multivariate analysis, Black patients and non-Black patients had similar PFS (UVA HR: 0.92, 95% CI, 0.63-1.33, *P* = .655; MVA HR 0.65, 95% CI, 0.38-1.1, HR *P* = .106), displayed in Table S2 and Table 4. Median PFS, 6-month PFS, and 12-month PFS were similar between non-Black and Black cohorts on Kaplan-Meier analysis in Figure 1D. In the White and other racial group cohort, median PFS was 6 months (95% CI, 4-8), while in the Black cohort, median PFS was 7 months (95% CI, 5-8). No differences in PFS by median household income by zip code were observed.

**Table 4.** Multivariate analysis of progression-free survival by racial subgroup.

		Progression-free survival time (months)		
	
Covariate	Total *N* = 110^a^	Hazard ratio (95% CI)	HR *P*-value	Type 3 *P*-value
**Racial group**	Black	0.65 (0.38-1.10)	.106	.106
	White and other racial groups	-	-	
**Baseline alkaline phosphate**	<102.50	0.61 (0.33-1.10)	.100	.100
	≥102.50	-	-	
**Baseline prostate-specific antigen**	<68.37	0.69 (0.38-1.24)	.210	.210
	≥68.37	-	-	
**Number of prior lines of therapy**		0.92 (0.80-1.05)	.219	.219
**CHAARTED trial result**	High	2.41 (0.80-7.24)	.117	.117
	Low	-	-	
**Gleason grade group**	6-7	2.83 (0.74-10.78)	.128	.241
	8	0.69 (0.11-4.16)	.683	
	9-10	0.28 (0.02-5.03)	.391	
	≤6	3.45 (0.47-25.31)	.224	
	Unknown	-	-	
**Total Gleason score**		2.61 (0.73-9.30)	.139	.139
**Number of metastases before lutetium-177–PSMA-617 treatment**		1.45 (1.13-1.86)	.004^*^	.004^*^
**Number of bone lesions at initiation**	1-19	2.00 (0.82-4.88)	.129	.129
	>20	-	-	
**ECOG at time of starting lutetium-177–PSMA-617**	0-1	0.68 (0.41-1.14)	.148	.148
	2+	-	-	

a Number of observations in the original data set = 163. Number of observations used = 110. *Patients who had a higher number of metastatic lesions prior to initiation of ^177^Lu-PSMA-617 had a significantly higher risk of disease progression.

### Overall survival

The entire cohort had a median OS of 15 months (95% CI, 13-22) with a 6-month OS of 75.6% (95% CI, 67.5%-81.9%) and a 12-month OS of 60.7% (95% CI, 51%-69.2%), displayed in Figure 1A. OS did not significantly differ between Black and non-Black cohorts, but displayed a trend toward longer survival among Black patients on univariate and multivariate analysis (UVA HR = 0.82, 95% CI, 0.49-1.37, *P* = .446; MVA HR = 0.59, 95% CI, 0.33-1.07, *P* = .08) as displayed in Table S3 and Table 5. The following variables were removed from the multivariate model using backward selection with an alpha level of removal of 0.25: patient age, baseline alkaline phosphatase, number of bone lesions at treatment initiation, prior taxane treatment, and Gleason score. On Kaplan-Meier analysis, median survival was 18 months (95% CI, 9-NA) for Black patients, compared to 14 months (95% CI, 11-NA) for White patients and patients of other racial groups, displayed in Figure 1B. OS was not significantly associated with median household income.

**Table 5.** Univariate analysis of overall survival.

		Overall survival time (months)		
	
Covariate	Total *N* = 151^a^	Hazard ratio (95% CI)	HR *P*-value	Type 3 *P*-value
**Racial group**	Black	0.59 (0.33-1.07)	.081	.081
	White/other	-	-	
**Median household income by zip code**	$0-$49, 000	0.62 (0.22-1.72)	.355	.191
	$100, 000-$150, 000+	1.08 (0.52-2.21)	.842	
	$50, 000-$74, 999	0.51 (0.23-1.10)	.086	
	$75, 000-$99, 999	-	-	
**Baseline prostate-Specific Antigen**	<68.37	0.32 (0.17-0.59)	**<.001** ^*^	**<.001**
	≥68.37	-	-	
**Number of prior lines of therapy**		0.87 (0.74-1.02)	.081	.081
**CHAARTED trial result**	High	14.19 (1.88-107.20)	**.010** ^**^	**.010**
	Low	-	-	
**Gleason score**	6-7	2.43 (1.09-5.45)	**.031** ^***^	.107
	8	1.27 (0.54-2.96)	.581	
	9-10	1.03 (0.44-2.38)	.947	
	≤6	2.08 (0.70-6.21)	.189	
	Unknown	-	-	
**Number of metastases before lutetium-177–PSMA-617**		1.70 (1.28-2.26)	**<.001** ^****^	**<.001**
**ECOG at time of starting lutetium-177–PSMA-617**	0-1	0.51 (0.28-0.95)	**.033** ^*****^	**.033**
	2+	-	-	

a Number of observations in the original data set = 163. Number of observations used = 151.

* Patients with lower baseline prostate-specific antigen have a decreased mortality risk.

** Patients with high-volume disease criteria per the CHAARTED trial had a higher mortality risk compared to patients with low metastatic burden.

*** Patients with Gleason score 6-7 had a higher mortality risk compared to the unknown group (HR P=0.31). However, the overall effect of Gleason scores did not reach significance (Type 3 P=0.107).

**** Patients with more metastatic sites prior to initiation of 177Lu-PSMA-617 had a significantly increased hazard of death.

***** Patients with an Eastern Cooperative Oncology Group performance status of 0-1 had significantly lower risk of mortality than patients with a performance status of 2 or higher at the time of initiation of lutetium-177–PSMA-617.

## Discussion

Our study found similar OS and PFS between patients with mCRPC treated with ^177^Lu-PSMA-617 who self-identified as Black compared to White or other racial groups. Black patients had significantly higher odds of a PSA50 response compared to patients of other racial groups, and a non-significant trend toward prolonged OS and PFS. In 2025, an estimated 57,330 Black men will be diagnosed with prostate cancer, with 6,150 resulting deaths in the US, reinforcing the importance of assessing ^177^Lu-PSMA-617 outcomes in diverse populations.^15^ To our knowledge, our cohort includes the largest proportion of Black patients treated with ^177^Lu-PSMA-617 reported to date. With similar PFS and OS, and improved odds of a PSA50 response for Black patients, our results highlight the importance of expanding access to ^177^Lu-PSMA-617 within diverse patient populations.

Although Black men experience worse survival outcomes from prostate cancer, prior studies have shown improved responses for Black men following chemotherapy, ^223^Radium, immunotherapy, and androgen-directed therapies.^11^^,^^16–18^ The reasons for this improved response to systemic therapies remain poorly understood. Underrepresentation of Black patients in clinical trials limits research progress in this area and reinforces the need for diversity in clinical trials.^19^^,^^20^ In our cohort, PFS and OS are similar for Black and White patients and aligned with findings from large prospective studies and other real-world cohorts, as displayed in Table 6.^8^^,^^21–23^

**Table 6.** Comparison of Emory Winship Cancer Institute cohort with reported literature.

Study	Number of patients treated with ^177^Lu-PSMA-617	Institution and country	Prospective or real-world study design	Prostate-specific antigen response rate 50	Progression-free survival	Median overall survival	Adverse events	Citation
**Emory real-world cohort**	163	Emory Winship Cancer Institute; USA	Real-world	55%	6 months (95% CI, 5-8)	15 months (95% CI, 13-22)	18.2% CTCAE grade ≥ 3	Current study
**Emory subgroup of patients identifying as Black**	66	Emory Winship Cancer Institute; USA	Real-world	63.5%	7 months (95% CI, 5-8)	18 months (95% CI, 9-NA)	14.3% CTCAE grade ≥ 3	Current study
**Emory subgroup of patients identifying as White or another racial group**	97	Emory Winship Cancer Institute; USA	Real-world	49%	6 months (95% CI, 4-8)	14 months (95% CI, 11-NA)	23.8 CTCAE grade ≥3	Current study
**Sartor et al.—VISION trial**	529	Multi-center study; USA and Europe	Prospective	Not reported.	8.7 months (imaging based)	15.3 months	52.7% grade ≥3	Sartor et al.^5^
**Hofman et al.—TheraP**	98	Multi-center; Australia	Prospective	66%	5.1 months (95% CI, 3.4-5.7) (PSA progression, radiographic, start of new cancer treatment, or death)	19.1 months	33% grade ≥3	Hofman et al.^6^ ^,^ ^7^
**Morris et al.—PSMAFore**	227	Multi-center; North America and Europe	Prospective	64%	11.6 months (95% CI, 9.3-14.2) radiographic progression free survival	23.66 months (95% CI, 19.75-NE)	36% grade ≥3	Morris et al.^8^
**Moradi Tuchayi et al.**	99	University of San Francisco, California, USA	Real-world	60.6%	5.8 months (95% CI, 5.0-6.5), Prostate-specific antigen PFS	12.7 months	21% grade 3+ thrombocytopenia, 33% grade 3+ anemia	Moradi Tuchayi et al.^21^
**Gafita et al.**	76	Johns Hopkins Hospital, USA	Real-world	41%	4.1 months (95% CI, 2.0-6.2)	13.7 months (95% CI, 11.3-16.1)	17% total CTCAE 5.03 grade ≥3	Gafita et al.^22^
**Almuradova et al.**	165	Multi-center; Turkey	Real-world	50.6%	8.2 months	13.5 months	19.4% CTCAE 4.03 grade ≥3	Almuradova et al.^23^

The etiology of prostate cancer disparities is multifactorial, likely related to social and economic factors, health-care access, housing disparities, and lower rates of insurance.^24^ Higher rates of comorbidities among Black men, including cardiovascular disease, diabetes, and hypertension, may further impact prostate cancer survival.^25–27^ A large study reported similar prostate cancer-specific mortality among Black and non-Black patients after adjusting for factors including access to health care and standard of care treatment, supporting literature that highlights structural and socioeconomic contributors to racial disparities.^24^ Barriers to ^177^Lu–PSMA-617 include the high costs associated with treatment and PSMA PET/CT imaging, corresponding insurance coverage, and access to tertiary centers that administer ^177^Lu–PSMA-617, which may disproportionately affect minority racial groups and low-income households.^28–30^ Efforts should focus on expanding access and insurance coverage for ^177^Lu–PSMA-617 across racial and socioeconomic groups.

Our cohort, in which 40.5% self-identified as Black or African American, and 49% self-identified as White, is more representative of Black patients than large, national registries. A study using a national database from the United States Department of Veterans Health Administration database of patients with mCRPC treated with abiraterone or enzalutamide identified 27% of patients as Black and 73% of patients as White.^31^ A study using the Flatiron Health national database reported 68.7% were non-Hispanic White and 10.6% were Black.^32^ In addition to increased racial diversity, our cohort better emulates a real-world population, including patients who often are older, have greater disease burden, and have more comorbidities compared to those enrolled in clinical trials.^33^ In our cohort, 83.9% of patients received prior taxane therapy, and those deemed unfit for taxane therapy due to high ECOG status, advanced age, fragility, or other comorbidities were offered ^177^Lu-PSMA-617. In comparison, in the VISION trial, all patients had received prior taxane treatment. Our study used a broad PFS definition reflecting real-world clinical endpoints, including PSA progression, imaging-based progression, death, or hospice transition, compared to the VISION trial, which used imaging-based PFS alone.^5^ Our real-world data suggest ^177^Lu-PSMA-617 is effective outside clinical trial settings and supports its earlier integration into mCRPC treatment regimens.

Limitations of our study include small cohort size, retrospective study design, and the use of binary racial categories. To mitigate selection bias inherent to single-institution studies, we included all patients who received at least one cycle of ^177^Lu-PSMA-617 and initiated treatment between July 20, 2022 and July 17, 2025, at Emory Winship Cancer Institute. Reliance on binary, self-reported racial categories restricted our ability to detect nuanced disparities within racial groups and does not account for the vast diversity that exists within racial groups. Our retrospective, single-institution analysis had a small number of participants from Hispanic, Asian, Native American, or Pacific Islander populations, which affected the study’s generalizability. We used census-level median income to approximate social determinants, although zip code proxies can systemically underestimate SES associations.^34^ Future studies using individual-level socioeconomic data may better capture these effects. Another limitation is the heterogeneity across institutions, the interpretation of nuclear medicine physicians, and the radiotracer used, which introduced variation in the PET imaging reading interpretations. In our analysis of PSMA PET/CT parameters, we included only Piflufolastat F-18 PSMA PET scans, despite some patients undergoing Gallium-68 PSMA scans. Due to variability in Gallium-68 PSMA scan quality and the growing use of Piflufolastat F-18 in the United States, we restricted our analysis to obtainable Piflufolastat F-18 PSMA PET scans. Radiographic and biochemical response discordance has been reported in approximately 25% of patients with mCRPC undergoing treatment.^35^ We did not collect data regarding radiographic response due to the retrospective nature of our study, but future prospective studies analyzing post-treatment would be beneficial to better characterize treatment responses. Despite our limitations, our cohort provides evidence supporting broader access to ^177^Lu-PSMA-617 to reduce prostate cancer disparities. Prospective validation of our findings in future studies is warranted.

## Supplementary Material

oyag022_Supplementary_Data

## Data Availability

The data supporting this article will be shared upon request by the corresponding author.
