# Supplementary material for: Clinical outcomes of lutetium-177–PSMA-617 in a racially diverse cohort of patients with metastatic castration-resistant prostate cancer
Source: Oncologist. 2026 Feb 3;31(3):oyag022. doi: 10.1093/oncolo/oyag022 (PMC12952921; doi:10.1093/oncolo/oyag022)
Supplement: oyag022_Supplementary_Data [file oyag022_supplementary_data.docx]

**Supplementary Materials**

**Supplementary Table S1. Univariate analysis of prostate specific antigen decline greater than or equal to 50% (PSA50)**

|  | | | **PSA 50=Yes** | | |
| --- | --- | --- | --- | --- | --- |
|  | | | **----------------------------------------** | | |
| **Covariate** | **Level** | **N** | **Odds Ratio (95% CI)** | **OR P-value** | **Type3 P-value** |
| Racial Group | White/Other Racial Groups | 87 | 0.56 (0.29-1.09) | 0.088 | 0.088 |
|  | Black | 63 | - | - |  |
| Median Household Income by Zip Code | $75,000 - $99,999 | 48 | 1.45 (0.48-4.36) | 0.513 | 0.080 |
|  | $50,000 - $74,999 | 41 | 3.80 (1.19-12.14) | **0.024** |  |
|  | $100,000 - $150,000+ | 43 | 1.98 (0.65-6.10) | 0.231 |  |
|  | $0 - $49,000 | 18 | - | - |  |
| CHAARTED Trial Result | Low | 21 | 1.76 (0.67-4.66) | 0.252 | 0.252 |
|  | High | 128 | - | - |  |
| Gleason Grade Group | Unknown | 40 | 1.58 (0.61-4.10) | 0.344 | 0.132 |
|  | <= 6 | 9 | 5.54 (0.98-31.25) | 0.052 |  |
|  | 9-10 | 48 | 2.64 (1.04-6.68) | **0.041** |  |
|  | 8 | 22 | 2.77 (0.90-8.58) | 0.077 |  |
|  | 6-7 | 31 | - | - |  |
| Taxane Treatment | Yes | 120 | 0.50 (0.19-1.31) | 0.158 | 0.158 |
|  | No | 22 | - | - |  |
| Number of Bone Lesions at Initiation | >20 | 113 | 0.97 (0.42-2.26) | 0.949 | 0.949 |
|  | 1-19 | 27 | - | - |  |
| ECOG at Time of Starting Lutetium-177–PSMA-617 | 2+ | 51 | 1.49 (0.74-2.97) | 0.263 | 0.263 |
|  | 0-1 | 94 | - | - |  |
| Patient Age |  | 150 | 1.04 (1.00-1.07) | **0.043** | **0.043** |
| Total Gleason Score |  | 113 | 1.18 (0.84-1.64) | 0.341 | 0.341 |
| Number of prior lines of Therapy |  | 148 | 0.88 (0.73-1.06) | 0.185 | 0.185 |
| Number of Lutetium-177–PSMA-617 Cycles |  | 150 | 1.97 (1.56-2.49) | **<.001** | **<.001** |
| Number of Metastases Before Lutetium-177–PSMA-617 |  | 144 | 0.73 (0.51-1.04) | 0.082 | 0.082 |

**Supplementary Table S2. Univariate analysis of progression-free survival**

|  | | | **Progression-Free Survival Time (Months)** | | |
| --- | --- | --- | --- | --- | --- |
|  | | | **----------------------------------------** | | |
| **Covariate** | **Level** | **N** | **Hazard Ratio (95% CI)** | **HR P-value** | **Log-rank P-value** |
| Racial Group | White/Other Racial Groups | 97 | 1.09 (0.75-1.58) | 0.655 | 0.633 |
|  | Black | 65 | - | **-** |  |
| Median Household Income by Zip Code | $75,000 - $99,999 | 50 | 0.79 (0.43-1.46) | 0.458 | 0.853 |
|  | $50,000 - $74,999 | 46 | 0.80 (0.43-1.49) | 0.486 |  |
|  | $100,000 - $150,000+ | 47 | 0.88 (0.48-1.62) | 0.679 |  |
|  | $0 - $49,000 | 19 | - | **-** |  |
| CHAARTED Trial Result | Low | 23 | 0.53 (0.29-0.97) | **0.038** | **0.024** |
|  | High | 138 | - | **-** |  |
| Gleason Grade Group | Unknown | 43 | 0.81 (0.48-1.38) | 0.443 | 0.770 |
|  | ≤ 6 | 11 | 0.60 (0.25-1.47) | 0.267 |  |
|  | 9-10 | 52 | 0.92 (0.55-1.55) | 0.750 |  |
|  | 8 | 23 | 0.83 (0.45-1.54) | 0.558 |  |
|  | 6-7 | 33 | - | **-** |  |
| Taxane Treatment | Yes | 129 | 1.19 (0.69-2.05) | 0.530 | 0.502 |
|  | No | 25 | - | **-** |  |
| Number of Bone Lesions at Initiation | >20 | 122 | 1.30 (0.80-2.12) | 0.287 | 0.252 |
|  | 1-19 | 30 | - | **-** |  |
| ECOG at Time of Starting Lutetium-177–PSMA-617 | 2+ | 54 | 1.49 (1.02-2.18) | **0.038** | **0.025** |
|  | 0-1 | 103 | - | **-** |  |
| Patient Age |  | 162 | 0.99 (0.97-1.01) | 0.278 | **-** |
| Total Gleason Score |  | 122 | 1.06 (0.87-1.28) | 0.585 | **-** |
| Number of prior lines of Therapy |  | 159 | 1.01 (0.92-1.12) | 0.785 | **-** |
| Number of Lutetium-177–PSMA-617 Cycles |  | 162 | 0.68 (0.61-0.75) | **<.001** | **-** |
| Number of Metastases Before Lutetium-177–PSMA-617 |  | 156 | 1.19 (0.98-1.45) | 0.078 | **-** |

**Supplementary Table S3. Univariate analysis of overall survival**

|  | | | **Overall Survival Time (Months)** | | |
| --- | --- | --- | --- | --- | --- |
|  | | | **----------------------------------------** | | |
| **Covariate** | **Level** | **N** | **Hazard Ratio (95% CI)** | **HR P-value** | **Log-rank P-value** |
| Racial Group | White/Other Racial Groups | 97 | 1.22 (0.73-2.05) | 0.446 | 0.437 |
|  | Black | 66 | - | **-** |  |
| Median Household Income by Zip Code | $75,000 - $99,999 | 50 | 0.71 (0.29-1.72) | 0.450 | 0.587 |
|  | $50,000 - $74,999 | 46 | 0.69 (0.28-1.68) | 0.410 |  |
|  | $100,000 - $150,000+ | 48 | 0.98 (0.42-2.31) | 0.971 |  |
|  | $0 - $49,000 | 19 | - | **-** |  |
| CHAARTED Trial Result | Low | 23 | 0.07 (0.01-0.52) | **0.009** | **<.001** |
|  | High | 139 | - | **-** |  |
| Gleason Grade Group | Unknown | 43 | 0.75 (0.37-1.55) | 0.444 | 0.676 |
|  | <= 6 | 11 | 0.99 (0.36-2.72) | 0.989 |  |
|  | 9-10 | 53 | 0.63 (0.31-1.28) | 0.202 |  |
|  | 8 | 23 | 0.97 (0.46-2.06) | 0.935 |  |
|  | 6-7 | 33 | - | **-** |  |
| Taxane Treatment | Yes | 130 | 1.06 (0.50-2.25) | 0.874 | 0.872 |
|  | No | 25 | - | **-** |  |
| Number of Bone Lesions at Initiation | >20 | 123 | 2.36 (1.11-5.01) | **0.026** | **0.020** |
|  | 1-19 | 30 | - | **-** |  |
| ECOG at Time of Starting Lutetium-177–PSMA-617 | 2+ | 55 | 1.92 (1.15-3.20) | **0.013** | **0.010** |
|  | 0-1 | 103 | - | **-** |  |
| Patient Age |  | 163 | 1.00 (0.97-1.03) | 0.940 | **-** |
| Total Gleason Score |  | 123 | 0.79 (0.62-1.01) | 0.056 | **-** |
| Number of prior lines of Therapy |  | 160 | 1.01 (0.88-1.17) | 0.879 | **-** |
| Number of Lutetium-177–PSMA-617 Cycles |  | 163 | 0.57 (0.49-0.67) | **<.001** | **-** |
| Number of Metastases Before Lutetium-177–PSMA-617 |  | 157 | 1.41 (1.09-1.83) | **0.010** | **-** |
